# Identification of aberrant microRNA expression pattern in pediatric gliomas by microarray

**DOI:** 10.1186/1746-1596-8-158

**Published:** 2013-09-20

**Authors:** Fatao Liu, Yuyu Xiong, Yang Zhao, Liming Tao, Zhou Zhang, Hong Zhang, Yun Liu, Guoyin Feng, Baojie Li, Lin He, Jie Ma, Shengying Qin, Yifeng Yang

**Affiliations:** 1 Institute for Nutritional Sciences, Shanghai Institutes for Biological Sciences, Chinese Academy of Sciences, 294 Taiyuan Road, Shanghai 200031, PR China; 2 Bio-X Institutes, Key Laboratory for the Genetics of Developmental and Neuropsychiatric Disorders (Ministry of Education), Shanghai Jiao Tong University, Shanghai 200030, PR China; 3 Shanghai Jiaotong University, Xinhua Hospital, No. 1665 Kongjiang Road, Shanghai 200092, PR China; 4 Institutes of Biomedical Sciences, Fudan University, Shanghai 200032, PR China; 5 Key Laboratory of Molecular Medicine, The Ministry of Education, Department of Biochemistry and Molecular Biology, Fudan University Shanghai Medical College, Shanghai 200032, PR China

**Keywords:** Pediatric gliomas, Pediatric brain tumor, MicroRNA, Expression, Microarray

## Abstract

**Background:**

Brain tumor remains the leading cause of disease-related death in children. Many studies have focused on the complex biological process involved in pediatric brain tumors but little is know about the possible role of microRNAs in the genesis of these tumors.

**Methods:**

In this study, we used a microRNA microarray assay to study the expression pattern of microRNAs in pediatric gliomas and matched normal tissues.

**Results:**

We found 40 differentially expressed microRNAs, among which miR-1321, miR-513b, miR-769-3p were found be related to cancer genesis for the first time. The expression of selected microRNAs were then confirmed by qRT-PCR. Furthermore, GO and pathway analysis showed that the target genes of the 40 differentially expressed microRNAs were significantly enriched in nervous system-related and tumor-related biological processes and signaling pathways. Additionally, an apoptosis-related network of microRNA–mRNA interaction, representing the critical microRNAs and their targets, was constructed based on microRNA status.

**Conclusions:**

In the present study we identified the changed expression pattern of microRNAs in pediatric gliamas. Our study also provides a better understanding of pediatric brain tumor biology and may assist in the development of less toxic therapies and in the search for better markers for disease stratification.

**Virtual slides:**

The virtual slide(s) for this article can be found here: http://www.diagnosticpathology.diagnomx.eu/vs/1323049861105720

## Introduction

Brain tumor, together with leukaemia, remains the leading cause of disease-related death in children [1]. According to a population-based study by Kaatsch and colleagues [2], approximately 60% of pediatric brain tumors are gliomas. Glioma is the most common type of primary brain tumor. Brain glioma can cause headaches, nausea and vomiting, seizures, and cranial nerve disorders as a result of increased intracranial pressure. Based on the observations that different gliomas share morphological similarities in different lineages of glial cells, the respective tumors have been classified as astrocytomas, oligodendrogliomas, and ependymomas [3]. However, because the pathogenesis of these tumors is unclear, treatment is particularly complex. Many children who have been treated for brain tumors experience significant long-term problems, such as changes in intellectual and motor function [4]. A better understanding of pediatric brain tumor pathogenesis is necessary to provide better markers for disease stratification and to assist in the development of less toxic therapies.

Aberrant microRNA expression has been found to be associated with a wide variety of human tumors, including lung cancer [5], cervical cancer [6], bladder cancer [7], esophageal adenocarcinoma [8] and pituitary adenomas [9] et al. MicroRNAs also play a significant role in brain tumor pathology by regulating target gene expression, apoptosis and autophagy [10]. The expression of miR-21 has been found to be increased between 5 and 100-fold in human glioblastoma tissues compared to control non-neoplastic brains [11]. A set of brain-enriched microRNAs, miR-128, miR-181a, miR-181b and miR-181c have been found to be down-regulated in glioblastoma [12]. Bottoni and his colleagues, using Northern blot, found that two microRNAs, miR-15a and miR-16-1 had a reduced expression in pituitary adenomas as compared to normal pituitary tissue [13]. Upregulation of mir-372 has also been found to be related with poor prognosis in glioma [14].

Large profiling studies using solid tissue and hematological tumors have established the usefulness of microRNA profiling for diagnosis and prognosis [15]. We therefore sought to determine the expression profiles of microRNAs in pediatric gliomas and matched normal tissues using microRNAs microarrays. We also performed Gene Ontology (GO) and pathway analysis to investigate the changed biological processes and signaling pathways involved in pediatric gliomas.

## Materials and methods

### Sample collection

For the study we recruited 8 patients undergoing surgery to treat astrocytomas at the XinHua Hospital. During surgery the tumor tissue and the matched adjacent noncancerous tissues were cut into small pieces and stored in liquid nitrogen. The tissues collected in XinHua Hospital were kept at −70°C until shipment to the Institute for Nutritional Sciences for RNA extraction and other experiments. Written informed consent was obtained from all patients or their representatives, and the Shanghai Committee of human rights approved the study. The general information of the tumor samples was summarized in Table 1.

**Table 1:** General information of the tumor samples involved in the present study

**No.**	**Patient No.**	**Gender**	**Age**	**Grade**
1	666088	M	4-year-old	WHO grade II
2	663136	M	2-year-old	WHO grade II-III
3	698211	F	4-year-old	WHO grade II
4	703380	M	1-year-old	WHO grade I-II
5	655390	M	12-year-old	WHO grade IV
6	688551	F	7-month-old	WHO grade IV
7	709578	M	10-month-old	WHO grade II-III
8	687901	M	5-year-old	WHO grade II

### MicroRNA microarray assay

#### Total RNA extraction and amplification

All of the RNA samples were extracted from tissues using the Trizol (Life technology) method. DNase I (New England Biolabs) was then added to digest the residual RNA. After purification, the concentration was measured using Nanodrop2000, all total RNA was reversely transcribed to cDNA using a MessageAmp™ II aRNA Amplification Kit (Ambion). T7 Enzyme Mix (Life technology) was then used to perform transcription of cDNAs to aRNAs in vitro. The linear amplified aRNAs were labeled using dissolved Mono-functional CyDye (Cy5) and purified by resin column.

#### Fluorescence labeling, hybridization and scanning

Firstly, microRNA probes (a total of 866 human microRNAs; miRBase, release 12.0) printed on the microRNA microarray (LC Sciences) were denatured at 95°C for 5 minutes. The microarray and probes were then hybridized with the Cy5-labeled aRNAs in the last step for 16 hours at 50°C. Next, 2X SSC + 0.2% SDS, 0.1% × SSC + 0.2% SDS, 0.1% × SSC (Amresco) were each used consecutively for ten minutes to wash the microarray. The microarray was then scanned and fluorescence signals were assessed.

#### Microarray data analysis

Data generated from the microarray was imported to Microsoft Excel. After normalizing the signal of each microRNA by using global average normalization as described by Bilban et al. [16], the expression level of each microRNA was calculated and student’s t test was performed to estimate between-group differences. For each microRNA, the difference between brain tumor and the matched adjacent noncancerous tissues was set to be significant if the fold change > 1.5 or < 0.67 and the p-value < 0.05.

### Quantitative RT-PCR analysis

Three of the up-regulated microRNAs, miR-1259, miR-21 and miR-222 and one of the down-regulated microRNAs, miR-128, were selected at representatives for verification. MiR-1259 and miR-128 exhibited lowest p-values, miR-21 was a well-known Onco-miR and miR-222 expression was also proved to be related with the development of a variety of tumors [17-20]. Expression of these microRNAs was assayed using stem-loop RT followed by PCR analysis as previously described (qRT-PCR) [21]. Real-time PCR was performed using TaqMan PCR kit (Life technology) on an Applied Biosystems 7900 Fast Real-Time PCR System (Applied Biosystems). In our study, qRT-PCR was performed in triplicate for each sample. The relative amount of microRNAs was normalized against U6 snRNA, the stability value of which was 0.046 according to Normfinder (http://www.multid.se/genex/hs410.htm). The fold change for each microRNA was calculated using the 2-[delta][delta] Ct method [22].

### Cluster analysis

We performed cluster analysis on the microRNAs that showed differential expression in brain tumors using Cluster (version 3.0, http://bonsai.hgc.jp/~mdehoon/software/cluster/software.htm) software. The TreeView tool (http://jtreeview.sourceforge.net/) was used to show the results generated by Cluster 3.0.

### MicroRNA target gene prediction

The sequences of microRNAs with differential expression were obtained from the Sanger microRNA Registry (http://www.sanger.ac.uk/software/Rfam/mirna). The sequences were used as query sequences to predict target genes on the UCSC human genome sequences and refgenes database (http://genome.ucsc.edu/). Only target genes that were expressed in the brain were retained.

### Gene Ontology (GO) analysis

We performed GO analysis on target genes of microRNAs with differential expression based on the Gene ontology database (http://www.geneontology.org/). Fisher’s two-side exact test and the Chi-square test were used to classify the GO categories, and the false discovery rate (FDR) was calculated to correct the P-value. We chose only GOs that had a p-value of <0.05 and a FDR of < 0.05.

### Pathway analysis

Pathway analysis was also performed on target genes of the differentially expressed microRNAs based on the KEGG database (http://www.genome.jp/kegg/). As in the GO analysis, two-side Fisher’s exact test and Chi-square test were used to classify the KEGG pathway categories, and the false discovery rate (FDR) was also calculated. Using a p-value cutoff of 0.05 and FDR cutoff of 0.05, we determined the enriched pathways.

## Results

### MicroRNA microarray analysis

**Figure 1:**
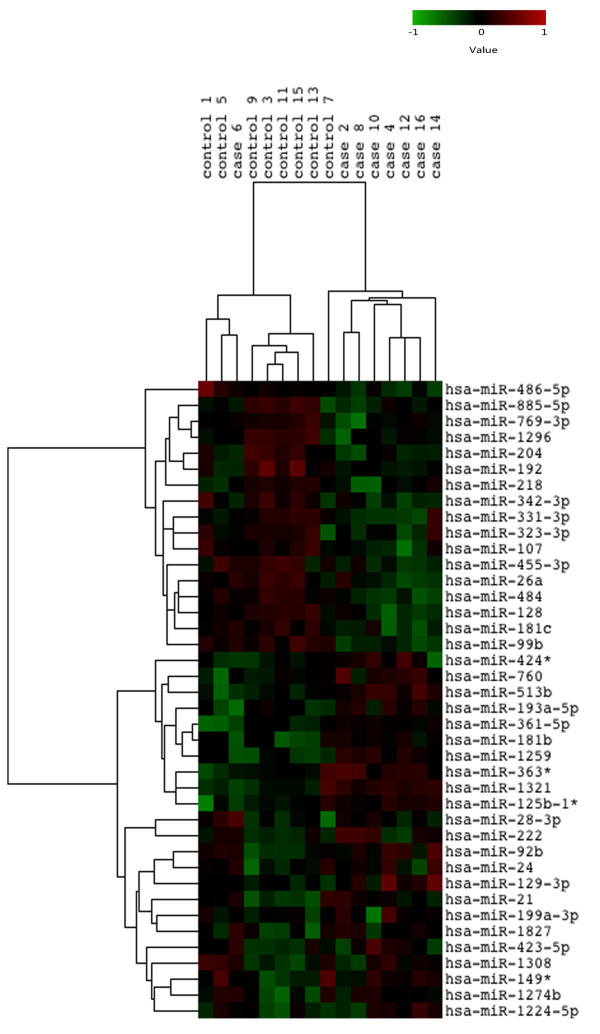
**Cluster analysis of the 40 differentially expressed microRNAs.** Results of cluster analysis indicate the changed microRNA expression pattern of brain tumors compared to the corresponding normal tissues.

In the microarray data analysis, we investigated more than 800 microRNAs in sum and found 40 differentially expressed microRNAs in gliomas compared to adjacent tissues. Among these, 23 up-regulated and 17 down-regulated microRNAs were involved. The information of the 40 microRNAs was summarized in Table 2. What needs illustration is that miR-24 was involved in the differential expressed microRNAs because it was with low p-value and FDR. Several recent findings suggested miR-24 played a role in the development of gliomas [23,24]. Thus, we still listed miR-24 in Table 2 considering the fold change (1.472878) was very closed to the threshold set by us. Cluster analysis was performed of the 40 microRNAs on the 8 brain tumors and corresponding adjacent noncancerous tissues. The results are shown in Figure 1.

**Table 2:** Characteristics of microRNAs that were significantly up- or down-regulated in pediatric brain tumors

**MicroRNA**	**P-value**	**FDR**	**Fold change**	**Genomic location**	**Strand**	**Region**
**Up-regulated microRNAs**						
hsa-miR-363^*^	0.007497	0.010704	10.427700	ChrX: 133303408-133303482	-	Intergenic
hsa-miR-181b	0.000527	0.002259	9.631896	Chr1: 198828002-198828111	-	Intron
hsa-miR-21	0.003809	0.008158	6.844031	Chr17: 57918627–57918698	+	Intergenic
hsa-miR-361-5p	0.000356	0.001831	6.274443	ChrX: 85158641-85158712	-	Intron
hsa-miR-1321	0.000636	0.002333	5.102661	ChrX: 85090785-85090863	+	Intergenic
hsa-miR-1259	0.000025	0.000650	4.581317	Chr20: 47896847-47896957	+	Intron
hsa-miR-222	0.023138	0.018584	3.929640	ChrX: 45606421-45606530	-	Intergenic
hsa-miR-92b	0.001809	0.005165	3.837460	ChrX: 85090785-85090863	+	Intergenic
hsa-miR-1274b	0.027961	0.020354	3.745325	Chr19: 58024375-58024441	-	Intergenic
hsa-miR-129-3p	0.033490	0.021517	3.154050	Chr7: 127847925-127847996	+	Intergenic
hsa-miR-1827	0.015657	0.015330	2.974803	Chr12: 100583662-100583727	+	Intergenic
hsa-miR-125b-1^*^	0.009024	0.011043	2.731521	Chr11: 121970465-121970552	-	Intron
hsa-miR-149^*^	0.041545	0.023727	2.409994	Chr2: 241395418-241395506	+	Intron
hsa-miR-760	0.003237	0.007563	2.151964	Chr1: 94312388-94312467	+	Intergenic
hsa-miR-424^*^	0.032166	0.021374	2.039437	ChrX: 133680644-133680741	-	Exon
hsa-miR-151-3p	0.013972	0.014363	1.884168	Chr8: 141742663-141742752	-	Intron
hsa-miR-193a-5p	0.023719	0.018584	1.830744	Chr17: 29887015-29887102	+	Intergenic
hsa-miR-513b	0.047111	0.025222	1.777760	ChrX: 146280562-146280645	-	Intergenic
hsa-miR-1224-5p	0.036530	0.022353	1.771082	Chr3: 183959193-183959277	+	Intron
hsa-miR-1308	0.039140	0.023393	1.737868	ChrX: 22080259-22080312	-	Intergenic
hsa-miR-423-5p	0.020774	0.018410	1.689889	Chr17: 28444097-28444190	+	Intron
hsa-miR-28-3p	0.017516	0.016077	1.527087	Chr3: 188406569-188406654	+	Intron
hsa-miR-24	0.032436	0.021374	1.472878	Chr9: 97848303-97848370	+	Intron
**Down-regulated microRNAs**						
hsa-miR-128	0.000356	0.001831	0.193099	Chr2: 136422967-136423048	+	Intron
hsa-miR-885-5p	0.006532	0.009875	0.266100	Chr3: 10436173-10436246	-	Intron
hsa-miR-99b	0.002762	0.005834	0.295771	Chr19: 52195865-52195934	+	Intron
hsa-miR-204	0.011402	0.012740	0.317649	Chr9: 73424891-73425000	-	Intron
hsa-miR-1296	0.006368	0.009875	0.351976	Chr10: 65132717-65132808	-	Intron
hsa-miR-455-3p	0.012976	0.013895	0.361298	Chr9: 116971714-116971809	+	Intron
hsa-miR-486-5p	0.006404	0.009875	0.379200	Chr8: 41517959-41518026	-	Intron
hsa-miR-218	0.028405	0.020354	0.382556	Chr4: 20529898-20530007	+	Intron
hsa-miR-192	0.009599	0.011213	0.411599	Chr11: 64658609–64658718	-	Intergenic
hsa-miR-323-3p	0.042741	0.023879	0.510200	Chr14: 101492069-101492154	+	Intergenic
hsa-miR-331-3p	0.016105	0.015330	0.515895	Chr12: 95702196-95702289	+	Intergenic
hsa-miR-769-3p	0.023862	0.018584	0.530823	Chr19: 46522190-46522307	+	Intergenic
hsa-miR-181c	0.021556	0.018466	0.540432	Chr19: 13985513-13985622	+	Intergenic
hsa-miR-342-3p	0.008581	0.011027	0.544354	Chr14: 100575992-100576090	+	Intron
hsa-miR-484	0.005505	0.009875	0.566546	Chr16: 15737151-15737229	+	3’ UTR
hsa-miR-107	0.028512	0.020354	0.609175	Chr10: 91352504-91352584	-	Intron
hsa-miR-26a	0.034746	0.021780	0.641470	Chr3: 38010895-38010971	+	Intron

The version of human genome reference assemble used in the study was GRCh37/hg19. * represents the opposite arm of the miRNA precursor.

### MicroRNA expression validation

**Figure 2:**
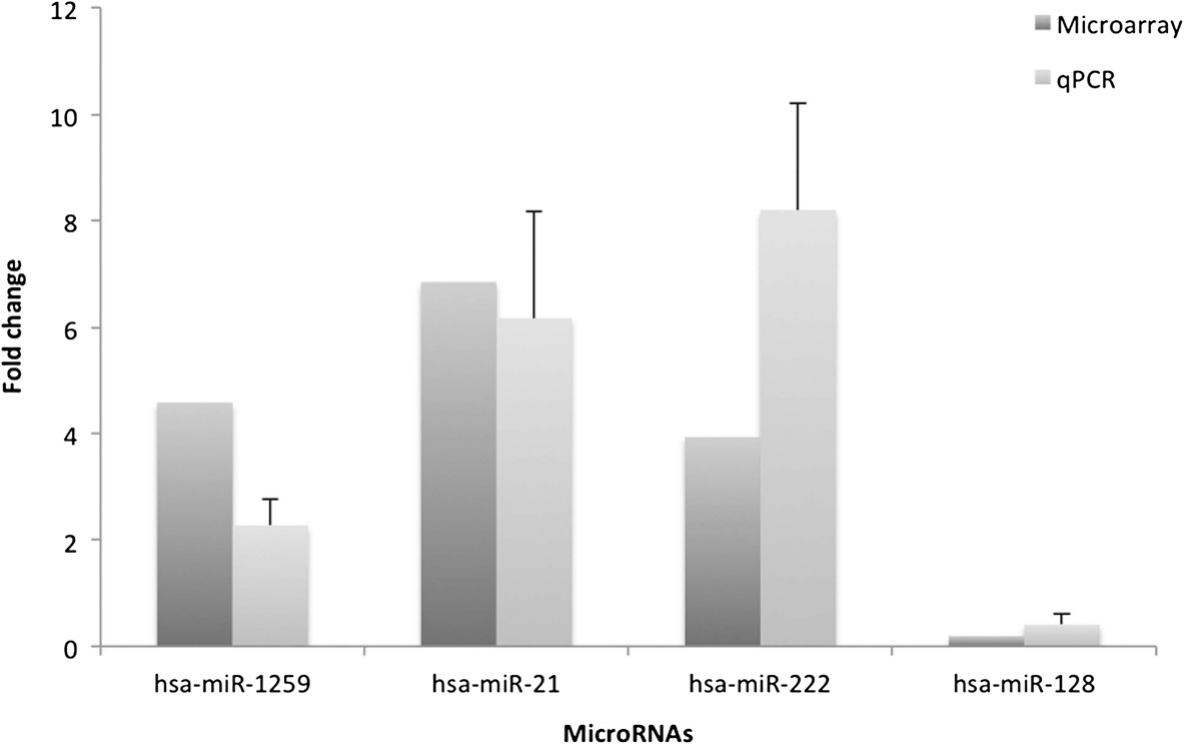
**Validation of microRNA expression by qRT-PCR.** The qRT-PCR assay shows the expression level of 4 microRNAs. qRT-PCR indicated that the fold change of microRNAs expression level in brain tumors was closely correlated with those detected by microarray.

In order to validate the microarray platform, we confirmed the expression of four randomly selected microRNAs that were strongly up- or down-regulated using qRT-PCR, using the same RNA samples that were used for the microarrays. The expression level of miR-1259, miR-21, miR-222 and miR-128 was identified. The fold change of each microRNA in tumors was calculated. The qRT-PCR results showed that miR-1259, miR-21 and miR-222 were up-regulated and that miR-128 was down-regulated, which was in line with the data generated from the microarray (Figure 2).

### Character analysis of MicroRNA target genes

We determined the target genes of each microRNA based on the UCSC Genome Browser Database. 2276 target genes of up-regulated microRNAs and 2403 target genes of down-regulated microRNAs were found to be expressed in the human brain. We then performed GO analysis on target genes of up- and down-regulated microRNAs, respectively. We found 143 significant GO categories for target genes of up-regulated microRNAs and 168 significant GO categories for target genes of down-regulated microRNAs.

**Figure 3:**
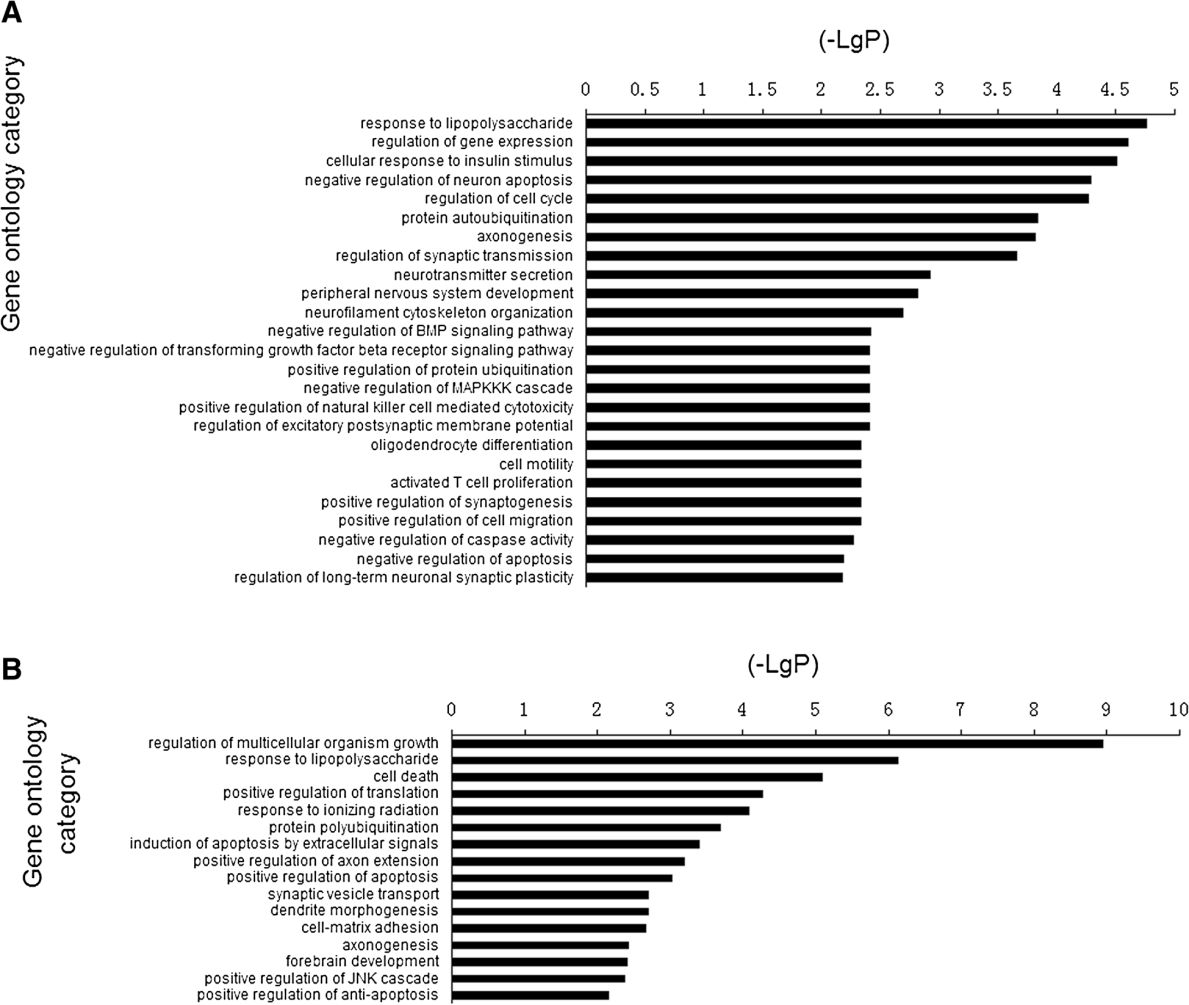
**GO categories based on biological processes for target genes of dysregulated microRNAs. (A)** Target genes of the up-regulated microRNAs were involved in negative regulation of neuron apoptosis, axonogenesis, regulation of synaptic transmission, neurotransmitter secretion et al. **(B)** Target genes of the down-regulated microRNAs were involved in axonogenesis, synaptic vesicle transport, positive regulation of axon extension et al.

Negative regulation of neuron apoptosis, axonogenesis, regulation of synaptic transmission, neurotransmitter secretion et al. were involved in the enriched GO categories of target genes of up-regulated microRNAs (Figure 3A), and the target genes of down-regulated microRNAs were enriched in synaptic vesicle transport, positive regulation of axon extension and positive regulation of apoptosis et al. (Figure 3B).

**Figure 4:**
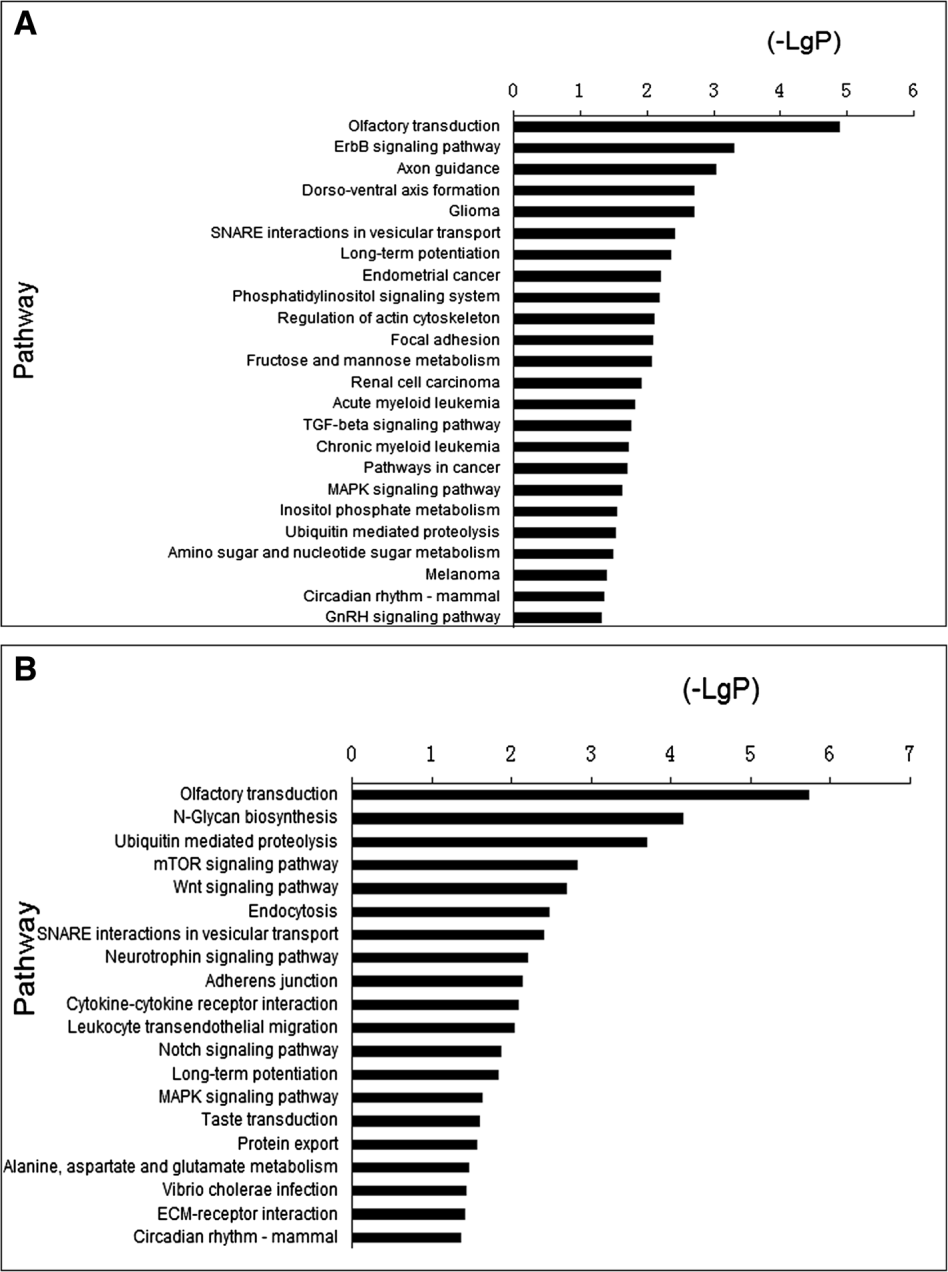
**KEGG pathway analysis for target genes of dysregulated microRNAs. (A)** Target genes of up-regulated microRNAs were involved in the ErbB signaling pathway, axon guidance, glioma, long-term potentiation, the MAPK signaling pathway et al. **(B)** Target genes of down-regulated microRNAs were related to the neurotrophin signaling pathway, the notch signaling pathway, amino acids metabolism et al.

Pathway analysis based on the KEGG pathway database was also applied. Using a statistical method similar to that in the GO analysis, we identified the enriched pathways of the target genes. From our data, target genes of up-regulated microRNAs were involved in the ErbB signaling pathway, axon guidance, glioma, long-term potentiation, pathways in cancer, the MAPK signaling pathway et al. (Figure 4A). Target genes of down-regulated microRNAs were related to N-Glycan biosynthesis, the Wnt signaling pathway, the neurotrophin signaling pathway, the notch signaling pathway, amino acids metabolism et al. (Figure 4B).

**Figure 5:**
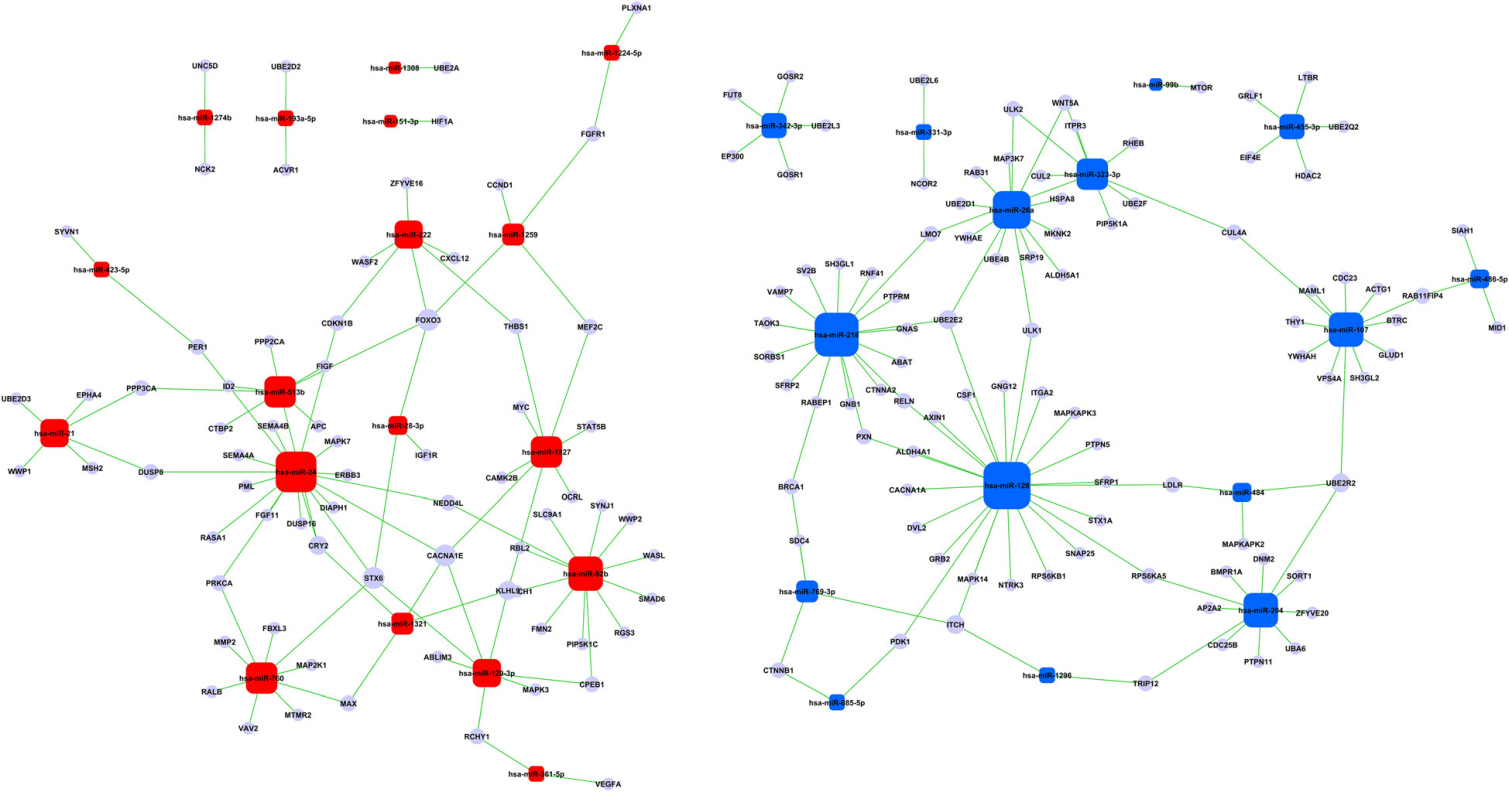
**MicroRNA-gene network analysis.** Target genes of the dysregulated microRNAs are assembled in the network according to their microRNA status. The circles represent genes that were involved in both the enriched GO categories and KEGG pathways. The squares represent the dysregulated microRNAs (red for those up-regulated and blue for those down-regulated). Green lines mark the interactions between microRNAs and corresponding target genes.

A total of 165 target genes involved in both the enriched GO categories and KEGG pathways were assembled in a microRNA-gene network, in which the interaction between microRNAs and their corresponding target genes was displayed. The microRNAs that had crucial roles in regulating the related biological processes and pathways were identified. Among the up-regulated microRNAs, miR-24, miR-92b and miR-760 were the top three key microRNAs in the network. The top three microRNAs in the down-regulated microRNAs were miR-128, miR-218 and miR-26a. As for target genes, CACNA1E (Voltage-dependent R-type calcium channel subunit alpha-1E), FOXO3 (Forkhead box protein O3) and STX6 (Syntaxin-6) were the top three key genes in the microRNA-gene network (Figure 5).

## Discussion

In the present study we used a microRNA microarray assay to study the differential expression pattern of microRNA in pediatric gliomas and the matched adjacent noncancerous tissues. Among the microRNAs detected in the microarray, we identified 40 microRNAs showing significantly higher or lower expression levels in tumors compared to the matched normal tissues. Results of GO analysis and KEGG pathway analysis suggested that target genes were closely associated with nervous system-related and tumor-related biological processes and signaling pathways.

Some of the microRNAs that showed differential expression between brain tumors and normal tissues in our study had previously been studied or found to be associated with gliomas, including pediatric gliomas. MiR-21, which was discovered to be upregulated by us, was also found and validated to be with increased expression in pediatric pilocytic astrocytoma (PA), a World Health Organization grade I pediatric glioma, in the study of Ho CY et al. [25]. Overexpression of miR-222 in gliomas was also observed by the research of Li Q et al. [26]. They proved that miR-222 could regulate Wnt/β-catenin signaling pathway and promote glioma genesis by using RNA interference and western blot technology. Furthermore, downregulation of miR-204, which was found to be associated with gliomas in our study, was also proved to contribute to glioma migration by targeting the migration-promoting receptor EphB2 [27]. In the present study miR-218 was discovered to be with decreased expression in pediatric gliomas, indicating the reverse relationship between miR-218 expression and development of gliomas. This was consistent with the findings of Tu Y et al. [28]. They found that upregulation of miR-218 reduced the migration, invasion and proliferation of glioma cells dramatically by regulating a wide range of genes and pathways. A new target gene of miR-218, which was noticed to be down-regulated in glioma in our study, has recently been found in the study of Shi ZM et al. [29]. They identified p70S6K1 as a novel direct target of miR-128 and overexpression of p70S6K1 can partly rescue the inhibitory effect of miR-128 in the glioma cells. This result further confirms our findings.

As expected, some of the microRNAs that showed differential expression in gliomas in our data were novel. There is little evidence about the relationship between miR-1321, miR-513b, miR-769-3p and cancer genesis. In addition, the upregulated microRNAs including miR-424^*^, miR-760, miR-513b, miR-361-5p, miR-1259, miR-363, miR-199a, miR-1827, miR-423-5p, miR-1308, miR-1274, miR-1224, miR-513b and the downregulated microRNAs including miR-885, miR-769, miR-1296, miR-192, miR-331-3p, miR-484, miR-99b were found to be related with gliomas for the first time. This may provide new clues for gliomas research.

Aberrant expression of microRNAs may contribute to the induction of pediatric gliomas by regulating genes involved in nervous system-related and tumor-related biological processes and signaling pathways. According to the result of GO analysis, the target genes were involved in negative regulation of neuron apoptosis, axonogenesis, regulation of synaptic transmission, neurotransmitter secretion and synaptic vesicle transport et al.. These biological processes are crucial for maintaining normal functioning of the nervous system. Alteration of the processes induced by aberrant expression of certain microRNAs may favor glioma-genesis. As for biological pathways, several cancer-related pathways, including glioma, endometrial cancer, the TGF-beta signaling pathway, the MAPK signaling pathway, the wnt signaling pathway and the notch signaling pathway et al. were among the enriched pathways of the target genes. TGF-beta signaling has long been considered to contribute to glioma pathogenesis by direct support of tumor growth, self-renewal of glioma initiating stem cells and the inhibiting of anti-tumor immunity [30]. Wnt signaling and MAPK signaling also play significant roles in almost all kinds of tumors, including glioma [31]. In recent studies, notch signaling has been proved to be dysregulated in brain tumors and to contribute to the malignant potential of these tumors [32]. Peng Xu et al. found out the different roles of Notch1 and Notch2. They proved that both upregulating of Notch1 and knocking down Notch2 had the effect of suppressing glioma cell growth and invasion as well as inducing apoptosis [33].

In conclusion, we investigated the changed expression patterns of microRNAs in pediatric gliomas and identified the possible role of microRNA and corresponding target genes in brain tumor pathology. However, due to the limitation of the microarray assay, only 866 known human microRNAs were detected. The role of more microRNAs in brain tumors needs to be investigated using more advanced techniques, such as second-generation sequencing and higher capacity microarrays. The present study does at least provide new insights into pediatric brain tumor biology and may assist in finding new diagnostic and therapeutic tools for these tumors.

## Conclusions

The changed expression pattern of microRNAs in pediatric gliomas was investigated and it may assist in finding new diagnostic and therapeutic strategies for these tumors. The specific microRNA expression pattern provides biomarkers for the diagnosis, staging and prognosis of gliomas. The differential expressed microRNA may also provide targets for drug development. MicroRNA mimics and inhibitors can strengthen and weaken the regulation of target genes respectively and may be novel antitumor candidates.

## Competing interests

The authors declare that they have no competing interests.

## Authors’ contributions

JM, SYQ and YFY: conceived of the study and helped to draft the manuscript. FTL, YYX, YZ, LMT and HZ carried out part of the experiments. FTL, LMT, ZZ and YFY performed the statistical analysis. FTL wrote the manuscript. YL, GYF, BJL and LH participated in the design and coordination of the study. All authors read and approved the final manuscript.
